# Contextual Factors Relevant to Implementing Social Risk Factor Screening and Referrals in Cancer Survivorship: A Qualitative Study

**DOI:** 10.5888/pcd21.230352

**Published:** 2024-04-04

**Authors:** Joseph A. Astorino, Mandi L. Pratt-Chapman, Laura Schubel, Judith Lee Smith, Arica White, Susan A. Sabatino, Robin Littlejohn, Bryan O. Buckley, Teletia Taylor, Hannah Arem

**Affiliations:** 1 The George Washington Cancer Center, The George Washington School of Medicine and Health Sciences, The George Washington University, Washington, District of Columbia; 2 Healthcare Delivery Research, MedStar Health Research Institute, Washington, District of Columbia; 3 Division of Cancer Prevention and Control, National Center for Chronic Disease Prevention and Health Promotion, Centers for Disease Control and Prevention, Atlanta, Georgia; 4 Department of General Medicine, Georgetown University, Washington, District of Columbia; 5 Howard University, Washington, District of Columbia; 6 Department of Oncology, Georgetown University, Washington, District of Columbia

## Abstract

**Introduction:**

Social risk factors such as food insecurity and lack of transportation can negatively affect health outcomes, yet implementation of screening and referral for social risk factors is limited in medical settings, particularly in cancer survivorship.

**Methods:**

We conducted 18 qualitative, semistructured interviews among oncology teams in 3 health systems in Washington, DC, during February and March 2022. We applied the Exploration, Preparation, Implementation, Sustainment Framework to develop a deductive codebook, performed thematic analysis on the interview transcripts, and summarized our results descriptively.

**Results:**

Health systems varied in clinical and support staff roles and capacity. None of the participating clinics had an electronic health record (EHR)–based process for identifying patients who completed their cancer treatment (“survivors”) or a standardized cancer survivorship program. Their capacities also differed for documenting social risk factors and referrals in the EHR. Interviewees expressed awareness of the prevalence and effect of social risk factors on cancer survivors, but none employed a systematic process for identifying and addressing social risk factors. Recommendations for increasing screening for social risk factors included designating a person to fulfill this role, improving data tracking tools in the EHR, and creating systems to maintain up-to-date information and contacts for community-based organizations.

**Conclusion:**

The complexity of cancer care workflows and lack of reimbursement results in a limited ability for clinic staff members to screen and make referrals for social risk factors. Creating clinical workflows that are flexible and tailored to staffing realities may contribute to successful implementation of a screening and referral program. Improving ongoing communication with community-based organizations to address needs was deemed important by interviewees.

**Summary**

**What is already known on this topic?**

Interest in the relationship between social risk factors and health care outcomes, including the ways in which social risk factors can negatively affect the cancer experience, is growing. However, research on how to implement social care interventions, particularly in oncology settings, is limited.

**What is added by this report?**

We studied how social risk factor screening and supportive care might be introduced for cancer survivors across various oncology settings.

**What are the implications for public health?**

Careful attention to staffing, availability of community referral partners, and electronic health record capabilities in oncology settings should be assessed before social risk factor screening and referral interventions are implemented.

## Introduction

Social determinants of health, or the conditions where we live, grow, work, age, and play, affect access to medical care and lead to disparate health outcomes in the US (1). A body of literature has emerged that focuses on implementing interventions in medical settings to assess and address social risk factors (individual-level factors such as lack of transportation to medical appointments, financial strain caused by medical bills, and food insecurity), including a 2019 social care report by the National Academies of Sciences, Engineering, and Medicine (2–4). Research has largely examined screening for social risk factors and referrals in emergency departments (5) and pediatric (6) and primary care (7) settings. This body of evidence has generated useful knowledge about validating tools to assess social risk factors (8), adjusting clinical care based on social context (9), integrating data on social risk factors into electronic health records (EHRs) (10), using referral technologies (11), aligning with community resources (12), and promoting advocacy by clinical and community partners to improve access to resources (13). Less research has examined the perceptions and experiences of clinicians and clinical staff members with integrating assessments of social risk factors into their workflows, including in oncology.

Cancer survivorship is a critical juncture in the cancer journey that is often not well defined in oncology settings focused on curative treatment. The number of cancer survivors is growing and was estimated at 18 million in 2022 (14). People with a history of cancer report higher levels of material (eg, affording care), psychological (eg, worry about affording care), and behavioral–financial hardships (eg, delaying or changing care plan due to cost) than people without cancer (15). A large body of literature demonstrates disparities in cancer-specific and overall survival outcomes by race, with worse outcomes among Black individuals compared with individuals of other races or ethnicities (16). This literature further suggests that strategies focused on upstream social determinants of health and downstream social risk factors are needed to increase equitable access to resources for improving patient quality and quantity of life.

The objective of this study was to describe the perspectives of clinical and support staff on workflows and contextual factors related to social risk factor screening in 3 oncology settings. This information, obtained through semistructured interviews, is intended to increase understanding of when and how to use existing oncology team structures to capture data on social needs and address them as patients complete cancer treatment. The interviews were the first phase of a multilevel research project aiming to reduce health disparities among people affected by cancer in Washington, DC, by implementing social risk factor screening and referral to community resources, providing community health worker support, conducting implicit bias training for health care professionals interacting with patients with cancer, and dismantling structural bias through system changes (17).

## Methods

In a series of key informant interviews, we explored current processes for identifying cancer survivors in each setting and current social risk factor screening processes. We used the Exploration, Preparation, Implementation, Sustainment (EPIS) framework (18) to guide the overall project. We sought to identify gaps in equitable care for individuals with a history of cancer, understand barriers and facilitators to delivery of supportive care, gather suggestions for delivering the proposed intervention, and prepare for sustainability after the project funding expires (18). To frame interview guides and analysis, we used the Exploration and Preparation phases, dually focusing on prevalent needs among cancer survivors and current workflows for oncology teams that might support or impede social risk factor screening. We further used EPIS to guide questions on topics in the inner setting (organizational characteristics and staffing, individual-level health care staff and provider characteristics), the outer setting (service environment or policies, funding, interorganizational environment, patient characteristics, and advocacy), bridging factors (partnerships with community-based organizations and community members), and linkages or relationships between EPIS topics. Although the exact questions varied depending on the role of the participant, the interview guide included key domains and questions across all interviews (Table 1).

**Table 1:** Interview Guide for Oncology Team Members in 3 Health Systems on Contextual Factors Relevant to Implementing Social Risk Factor Screening and Referrals in Cancer Survivorship, Washington, DC, February–March 2022

Domain	Example questions
Survivorship care and gaps	1. Please describe what happens at the end of curative treatment or the treatment plan (eg, watch and wait) for breast/prostate cancer. 2. What does survivorship care look like at your institution at present? What processes are in place to address nonmedical needs such as food insecurity, transportation, or insurance? 3. What are the most common issues/needs cited by cancer survivors? 4. What are the most common services used by cancer survivors?
Current screening practices	5. Do you use a particular screening tool during the transition to survivorship (eg, distress thermometer)? 6. Please describe any current screening processes such as for mental or physical side effects (eg, neuropathy, lymphedema, fatigue, distress) of treatments that are delivered to patients after completing treatment. 7. How have patients reacted to being screened for social risk factors?
Data collection infrastructure	8. Please describe what happens to the screener data after it is collected. a. Who receives the data (within or external to your organization)? b. Does the screener data get stored? If yes, please describe where and how the data are stored. c. Does the screener data get entered into the electronic health record (EHR)?
Referral processes	9. How do you identify the appropriate next step if a need is identified (eg, referral to social worker, community health organizations)? 10. What do you do if you do not know of a service that can meet the identified need? 11. How are patients’ refusals for needed and offered services documented? 12. Do you receive follow-up information if a patient has received a service for an identified need (ie, closed loop on referred patients)?
Preferences for an intervention	13. If you could implement any process for social risk factor screening and referring cancer survivors in your clinic, what would it look like? 14. What resources and/or technology would make the following processes easier? a. Identifying/flagging survivorship patients b. Screener delivery c. Referrals d. Service follow-up e. Social risk factor screening and service data tracking

### Site selection

We recruited participants from 3 health systems in Washington, DC, that serve a large proportion of cancer patients from historically marginalized groups, including patients with lower socioeconomic status and Black patients. Two sites offer specialized care by cancer site, while 1 site offers only general oncologists. Each site uses a different EHR system. Furthermore, 2 sites had implemented a new EHR system within the 2 previous years that included new forms for documenting social risk factors.

### Data collection

We used purposive sampling to identify clinical and support staff who worked with breast and prostate cancer patients at each site and reached out by email. We performed 18 semistructured interviews lasting approximately 45 to 60 minutes and recorded them via Microsoft Teams (https://www.microsoft.com) from February 3 to March 11, 2022. Each interview was transcribed automatically and then reviewed by our team for quality control. Final transcripts were uploaded into the qualitative software program Dedoose (SocioCultural Research Consultants, LLC) for analysis. The Georgetown–Howard Universities Center for Clinical and Translational Sciences institutional review board approved this study, and all interview participants provided informed consent.

### Analysis

Our team developed an a priori codebook based on the EPIS framework. Three team members (2 authors [J.A.A., R.L.] and a clinical research coordinator) independently coded 1 transcript from each setting and then compared initial codes. The study leader (H.A.) and all coders met to discuss discrepancies in the application of codes until consensus was reached. Adjustments were made to the codebook to better define codes and create new codes as needed, and all data were divided evenly among the 3 analysts and independently coded. We identified frequent codes and code co-occurrences, organized EPIS constructs that affected social risk factor screening into groups, and extracted representative quotations.

## Results

The 18 interview participants included clinical providers (oncologists, nurse practitioners, physician assistants, and nurse navigators) and patient support staff (social workers, patient navigators, and patient advocates). Nine participants identified as Asian, 5 as White, and 4 as Black; 11 participants were female (Table 2). Participants ranged in age from 28 to 72 years. The response rate, calculated as the number of individuals who responded divided by the number of individuals contacted by email, was 56% (18 of 32); 1 person declined, and the remainder did not respond to our outreach. We did not continue to pursue outreach or participation once we reached our target sample size (>15), and the team agreed that thematic saturation had been achieved. We found no indication that willingness to participate was affected by the participant’s role or affiliated institution. In alignment with research goals, we achieved representation across the various roles of the cancer care team. 

**Table 2:** Demographic Characteristics of Interview Participants (N = 18), Oncology Team Members in 3 Health Systems, Washington, DC, February–March 2022

Characteristic	No.
**Race**	
Asian	9
Black or African American	4
White	5
**Ethnicity **	
Non-Hispanic or Latino	18
**Sex**	
Female	11
Male	7
**Age, mean (range), y**	46 (28–72)
**Clinical role**	
Medical doctor, physician assistant, nurse practitioner	10
Registered nurse, social worker	5
Other patient support staff	3
**Cancer center**	
Site 1	4
Site 2	7
Site 3	7

EPIS constructs that affected social risk factor screening were organized into the following groups: 1) outer context: environment and policies, 2) inner context: organizational characteristics and organizational challenges, and 3) bridges, including relationships with social service organizations (Table 3). Emergent themes and example quotations are outlined in Table 4.

**Table 3:** Exploration, Preparation, Implementation, and Sustainment (EPIS) Framework–Relevant Factors Shaping Social Needs Screening and Referral Workflows in Cancer Survivorship: Summary of Results of Qualitative Interviews of Oncology Team Members (N = 18) in 3 Health Systems, Washington, DC, February–March 2022

EPIS domain	Construct	Examples from interviews
**Outer context**		
Environment	Existing organizations to handle social needs	Provider perceptions of availability of resources or experiences with referrals
	COVID-19	
Policies	Lack of reimbursement for social support services	Social workers and community health workers are typically grant funded
**Inner context**		
Organizational characteristics	Role specialization	Individuals seeing cancer survivors differed by institution (eg, medical doctor, nurse practitioner, registered nurse); social work typically did not receive new referrals during the survivorship phase
	Staffing	When there was staff turnover, there was limited capacity to cover that role and provide institutional knowledge
	Culture	Providers and patient support staff recognized a need to support cancer patients after completing curative treatment
Organizational challenges	Data tracking	Challenges to identifying survivors
		Screening and referral workflows relied on person-to-person conversations
		Overburdened providers did not have capacity for administering additional screenings
		Electronic health record capabilities differed by institutions
**Bridges between health care systems and community resources**		
Health care–community partnerships	Reliance on personal relationships between health care system staff and community-based organizations	Relationships were mentioned between individual employees more than between referring institutions; thus, when a staff member left an organization, the relationship was lost
Knowledge of resource availability	Challenges in maintaining up-to-date information on social services	Specialized staff suggested to fulfill this role

**Table 4:** Example Quotations Mapped to Exploration, Preparation, Implementation and Sustainment (EPIS) Domains: Results of Qualitative Interviews of Oncology Team Members (N = 18) in 3 Health Systems, Washington, DC, February–March 2022

Domain	Example quotation	Participant role
Outer context	When you . . . see housing as a problem area that they need help with, [it] is always just like . . . [I] can help you with transportation. I can try to get financial assistance depending on your cancer. But housing is kind of, like, almost impossible.	Setting 1, patient support staff
	[I]n DC, there is kind of, like, one-stop shops called core service agencies that provide . . . mental health [services]. They do psychiatry. Sometimes they will have, like, medical, like PCP [primary care physician] services and then . . . case management to just help you with all . . . the social welfare, finances, finding a job, you know, things like that . . . but it’s really only for people that have Medicaid.	Setting 3, patient support staff
	Now I’ve got [patients] who are talking about going to be evicted from their homes because of . . . COVID pandemic issues and all that kind of stuff coming to an end . . . so I think the big things that come up to me . . . on an ongoing basis, one is housing. Some people have, you know, under government . . . programs; they’re in hotels or affordable housing, but it’s sort of not secure.	Setting 3, oncologist
	[T]here was no sort of attestation that, like, I have, you know, sufficiently and thoroughly reviewed the patient’s social determinants of health. . . . [I]t may be there . . . in terms of comprehensive cancer care in the future. . . . We were addressing it, but not in sort of a formal way to get credit for it.	Setting 2, oncologist
	I don’t think . . . they have a way to screen any of them because [we] don’t have the staffing to be able to do all that.	Setting 1, oncologist
Inner context	[W]e don’t have any system . . . on the computer. [It] would be nice if we [had] this electronic . . . medical system. . . . [M]ost of, I think, what we have been doing is . . . communication . . . emails to say, hey, this is a patient that . . . finished the chemotherapy. . . . [H]e’s going to be followed by you, by me. . . . But I think [it] all depends on the . . . physicians. That’s exactly what we are doing now . . . lots of . . . burden to us.	Setting 2, oncologist
	And the great thing about [our center] is that we don’t work as an island. We have a resource center. . . . I collaborate with a nutritionist. I collaborate with the social workers . . . and financial navigators. So, a host of us are really working in collaboration to provide excellent care.	Setting 3, patient support staff
	[W]e definitely need . . . more of a team approach so that there’s not a gap when one person is not here.	Setting 2, patient support staff
	I don’t think I’ve ever really gotten a referral for a survivor needing services. Yeah, it would usually start with, they were in treatment, and then if there’s continued needs, then I would try to assist as best as I can.	Setting 3, patient support staff
Data tracking	I can say *nobody* ever refers to the distress screen when they . . . contact me. I think . . . maybe a handful of times they’ll be like . . . “[The] patient is saying they are seven or eight on the distress screen. Can you see them?” But usually, you know, when they refer it’s like they already know the specific need, and if they did use a distress screening to find that out, they don’t let me know that part.	Setting 3, patient support staff
	Who would I want to do [it]? . . . [The] nurse navigators . . . or I like the idea of medical assistant doing things, but if they don’t tell us the answers, it’s kind of useless. Like they need to tell us . . . “[O]h, this patient answered yes to this question,” and it needs to verbally come out of their mouth, because if they document it somewhere in the medical record, I probably will never see it.	Setting 3, patient support staff
Bridges between clinic and community	A lot of those [connections] have just come with . . . I’ve been in this area my whole life. So, I’ve really developed a lot of . . . support from people. And by them, knowing that I work here, it’s easy for me to call people.	Setting 1, patient support staff
Suggestions for implementation	[W]e’re able to get more details when we actually talk with the person, and I definitely don’t want to lose that piece of it, of the assessment, actually . . . having the conversation. I don’t want it to all be just like something they’re filling out on the iPad.	Setting 2, patient support staff
	In sort of thinking about . . . ways to . . . make . . . this area of care for patients really . . . sturdy is certainly increasing staffing . . . because I think that that’s been a challenge across many areas right now.	Setting 1, patient support staff
	I think one of the things that can be challenging is trying to search and find reputable resources. And when you find [them], you hold onto them, and that’s fantastic, but if there was . . . a database in which these places were already sort of vetted so that we know that they’re well equipped to support our patients, that would be fantastic.	Setting 2, patient support staff
	[T]hat information could just automatically go into [EHR] so that . . . anybody on the team could access it easily and it would all be in the patient’s chart, easily accessible. . . . I feel like that would be helpful. . . . Also, if there was some way that . . . we could indicate in the chart that we had made referrals or what we had done outside of just . . . typing it in a note.	Setting 2, patient support staff

### Overview of existing processes for social risk factor screening

None of the 3 institutions had systematic processes in place for social risk factor screening and referral for cancer survivors. Additionally, none had a distinct survivorship program: we found differences in how patients navigated through the end of curative treatment (defined as surgery, radiation, chemotherapy, or immunotherapy) and in the types of providers that were involved with the patient after completion of curative treatment. These different experiences affected the way interview participants responded to options for integrating universal social risk factor screening for individuals with a history of cancer into existing clinical workflows.

### Outer context

Outer context factors affecting social risk factor screening and referrals for cancer survivors described by interview participants centered on environment and policies. Perceptions of the social service resources available in Washington, DC, affected individual provider decisions on social risk factor screening and referral. Specifically, because of a lack of resources in the area, support staff were hesitant to ask questions about topics such as housing and mental health. Interview participants also described the challenge of keeping up with the eligibility criteria of community-based organizations and keeping track of where to refer patients.

Another environmental factor affecting social risk factors and the ability to address identified needs was the COVID-19 pandemic. The structure for team-based care was weakened by the COVID-19 pandemic. Some positions were eliminated, and members of oncology care teams were asked to take on additional roles and responsibilities. The pandemic affected options for transdisciplinary clinics, led to staffing shortages as burnout increased, and simultaneously exacerbated patient social risk factors, especially among patients with financial constraints.

Policy can also limit availability of resources for assessment of social risk factors during treatment. Participants noted that social needs were assessed primarily by using the National Comprehensive Cancer Network’s distress thermometer (19), a tool that documents a problem list only for people who first identify distress. The lack of reimbursement for work related to social risk factors affected providers’ perceptions of who might deliver screening and address identified needs. Providers indicated that they were not provided incentives to complete such screenings and had limited capacity to respond to any identified social needs. Without designated individuals responsible for conducting social risk factor screening, we found limited staffing options for conducting screening or providing patients with additional services.

### Inner context

Differences in cancer treatment trajectories by stage, cancer site, and clinic capacity meant that no single recommendation emerged for how to conduct social risk factor screening for patients as they complete treatment. We found no consensus across institutions or cancer sites about when survivorship topics are discussed with patients. No institution had a way to identify cancer survivors in the EHR. Rather, the institutions coordinated care after acute treatment through direct communication between providers and often through manual tracking.

The idea of cancer survivorship and how patients navigated oncology care after completion of acute treatment differed both by institution and cancer site. Discussion of survivorship with patients and timing of visits varied based on cancer stage, treatment type, and treatment duration or cadence. Some posttreatment pathways in oncology were shaped by institutional accreditation standards (eg, whether a survivorship care plan would be provided) and the availability of resources or staffing. Furthermore, the providers of “watch and wait” care for patients with prostate cancer were noted to be different from the providers of care for patients with late-stage disease. These differences complicate the process for tracking patients who are completing treatment because some care for prostate cancer is delivered in ambulatory settings rather than the hospital.

Across the 3 settings, where social risk factors were identified, advanced practitioners (physicians, physician’s assistants, and nurse practitioners) referred their patients with identified social needs (defined as social risk factors where a patient wanted help) to a primary point of contact in the cancer institute (eg, a nurse navigator or social worker) to better assess needs and provide the appropriate referral. Although navigators and social workers at some sites described opportunities to provide referrals or services for social needs, not all settings employed people in these roles, and all settings had limited capacity to provide active follow-up given high patient volumes. Social workers and support staff also described how oncology social work typically focuses on newly diagnosed patients or those in treatment rather than those who have completed treatment and largely depends on grant funding because these services are not billable. Still, support staff recognized the value of screening during survivorship to identify any emerging social risk factors, such as financial toxicity (a term used to describe the harmful effect of the high cost of treatment on a person’s quality of life) or social isolation.

### Data tracking

Challenges to implementing systematic social risk factor screening and referral included the recording of data on social risk factors in different formats, such as verbal exchanges, paper surveys, and EHR communications. Two institutions had migrated to a new EHR just before the start of this study, which created a host of competing priorities as the institutions learned their new systems. Interview participants also identified issues with ease in accessing documented patient information about social risk factors in the EHR, suggesting that electronic documentation alone would not improve information sharing. The process of tracking referrals and follow-up was especially challenging when the referrals were to an organization outside the health care system, rather than an internal referral to social work.

### Bridges from clinical to community settings

In some contexts, participants described a reliance on informal social networks for referrals to community organizations (ie, personal relationships where an individual would pick up the telephone and call a community-based organization), while in other contexts, staff relied on lists created by previous employees for identifying referrals. In both situations, health care professionals and support staff depended on the individual who had built and maintained those connections rather than a communal resource or database for referrals. While some participants made references to public access databases such as FindHelp (www.findhelp.org), most of the time staff preferred to refer patients to trusted resources where they knew that the patient would receive the promised supports.

### Suggestions for implementation

Despite considerable differences in processes for providing cancer survivorship care, some suggestions for implementation of social risk factor screening were similar across institutions. Many participants suggested better ways of tracking patients in the EHR as they completed treatment; better communication within care teams, including closing the loop on referrals; and building better bridges with community-based organizations. Also, interview participants suggested that additional staff, including someone with the specialized knowledge and ability to maintain resources, was needed to address identified social needs for patients rather than relying on existing staff with other responsibilities. Participants largely cautioned that self-administered screeners would increase burden and fatigue among patients and could detract from the personal touch of interacting with patients.

## Discussion

We found that providers and staff were aware of the importance of assessing and addressing social risk factors, but current resources and workflows did not support systematic efforts to assess or address social risk factors for breast and prostate cancer patients who were completing treatment. Still, opportunities were identified for improved processes, including better EHR capabilities and dedicated staffing.

The literature on delivering social risk factor screening and referrals is growing and includes a recent scoping review on provider knowledge, attitudes, and behaviors (20) and a 2022 comprehensive report on social risk factor evaluation, including information on workflows and perspectives of providers and patients (21). Studies generally have suggested that the level of knowledge among providers varies about how to screen and refer, including some provider discomfort in screening due to lack of training (22,23). The providers in our study did not report a lack of training, but they did report discomfort in asking social need questions that they perceived they could not address. Studies have shown that most providers endorse the importance of assessing and addressing social risk factors (24,25), similar to what we found. Even where providers recognize a need for social care, or services in a medical setting that address social needs (2), implementing such services requires a shift in mindset of what defines and determines quality health care and how it should be paid for (26,27). In our study, providers mentioned accreditation standards and reimbursement systems as influential in determining who could deliver screening and when they could in cancer care settings.

In alignment with existing literature that suggests that the COVID-19 pandemic increased awareness of social determinants of health and motivated the health profession to assess social risk factors in clinical settings (28), we found that participants mentioned increased awareness of the need for such screening and referral programs. However, we also found that the pandemic weakened the capacity to address social needs in cancer care settings. Redirected staff and clinician time, burnout, and turnover negatively affected staffing, clinic structures (eg, elimination of transdisciplinary clinics), and the availability of resources. Previous literature has suggested that clinicians would screen for social risk factors more often if they had more community-based resources to assist those in need (22). The degree to which these outer context environmental factors influence workflows should be considered when planning interventions, even if the inner context, or culture of the health care system, supports social risk screening and referrals.

While many of the described challenges to social risk factor screening exist across the cancer experience (ie, during prevention and treatment), the cancer survivorship period has its own special challenges. The 3 health care systems in our study, like many in the US, did not have standardized survivorship care programs and patients were not uniformly offered supportive care. Apart from physical late and long-term effects, cancer survivors experience work-related concerns, financial toxicity, and supportive care needs (29,30). Studies have documented associations between financial toxicity and worse quality of life (30). The literature describes survivorship care as a time where individuals are “lost in transition,” underscoring the importance of attending to the specific needs of cancer survivors after treatment (31,32).

Given differences observed among institutions and within cancer-site–specific clinics, tailoring programs for clinical context, including patient and provider characteristics and workflows (33), is critical for successful implementation (34). Studies have highlighted time constraints (35,36) and lack of integration into existing workflows (37) as major barriers to systematic screening for social risk factors. We also observed these barriers. The fit between social risk factor screening innovations and organizational contexts is difficult to assess, but previous attempts at implementing similar psychosocial assessments, such as the National Comprehensive Cancer Network’s distress thermometer (19), can serve as a useful indicator of potential for tailoring delivery in cancer care settings. Our results also demonstrated the complexity of finding a distinct time point to screen patients for social risk factors and refer to cancer survivorship resources. This complexity results from differences in patient treatment courses and differences in clinical resources. A recent scoping review that examined workflows for addressing social risk factors in ambulatory settings found that although settings varied in who could assess risk factors, 88% of published studies (57 of 65) reported using social care staff to address patient needs (38). This review supports our findings on preferences to minimize disruption to existing workflows by depending on specific support staff to provide appropriate referrals.

The process of standardizing data about social risk factors in structured databases, such as using Z codes (diagnosis codes for social risk factors from the *International Classification of Diseases, 10th Revision, Clinical Modification*) (39) and creating closed loop referrals, whereby providers can track whether a referral was completed and whether a need was addressed, is a growing area of research (40). The 3 institutions in our study cited the tracking of screening and referral information as a challenge. Barriers discussed by participants included a reliance on informal or ad hoc information exchange among providers, clinical staff, and community-based organizations. This information then became difficult to track through the EHR, especially during the process of changing EHR systems.

### Limitations

Limitations of our study included a small number of interview participants from setting 1, where resources were more limited and fewer staff were available for interviews, suggesting that we may have been unable to capture the diversity of perspectives on the integration of social care in oncology. Furthermore, interviews were conducted during the COVID-19 pandemic, which affected workflows and staffing and caused staff turnover. Still, a strength of our study was that we included providers from multiple professional roles across 3 settings.

### Conclusion

The process of implementing social risk factor screening and referral processes involves unique challenges for those who work in cancer survivorship care. Many health care systems have no way to identify who has completed treatment in a discrete field in the EHR; this knowledge generally comes from oncology teams tracking complex trajectories of care. However, social risk factor screening is widely recognized as critical for improving health outcomes and an opportunity for cancer centers to work toward health equity. Integrating social needs screening and referral technologies into care for cancer survivors will improve tracking capacities and linkages with community-based organizations. Participants in our study had many suggestions for how to implement these interventions and demonstrated practice-based knowledge that can be harnessed for tailoring efforts. Overall, additional staff support, alignment of accreditation requirements and payment models for clinic-based screening and referral, and EHR documentation of closed-loop referrals will be important for social needs interventions to be sustainable in clinical settings.
